# The combination of *Neosartorya* (*Aspergillus*) *fischeri* antifungal proteins with rationally designed γ-core peptide derivatives is effective for plant and crop protection

**DOI:** 10.1007/s10526-022-10132-y

**Published:** 2022-02-04

**Authors:** Liliána Tóth, Péter Poór, Attila Ördög, Györgyi Váradi, Attila Farkas, Csaba Papp, Gábor Bende, Gábor K. Tóth, Gábor Rákhely, Florentine Marx, László Galgóczy

**Affiliations:** 1 Institute of Plant Biology, Biological Research Centre, Eötvös Loránd Research Network, Temesvári krt. 62, 6726 Szeged, Hungary; 2 Department of Plant Biology, Faculty of Science and Informatics, University of Szeged, Közép fasor 52, 6726 Szeged, Hungary; 3 Department of Medical Chemistry, Faculty of Medicine, University of Szeged, Dóm tér 8, 6720 Szeged, Hungary; 4 MTA-SZTE Biomimetic Systems Research Group, University of Szeged, Dóm tér 8, 6720 Szeged, Hungary; 5 Department of Microbiology, Faculty of Science and Informatics, University of Szeged, Közép fasor 52, 6726 Szeged, Hungary; 6 Department of Biotechnology, Faculty of Science and Informatics, University of Szeged, Közép fasor 52, 6726 Szeged, Hungary; 7 Institute of Biophysics, Biological Research Centre, Eötvös Loránd Research Network, Temesvári krt. 62, 6726 Szeged, Hungary; 8 Institute of Molecular Biology, Biocenter, Medical University of Innsbruck, Innrain 80-82, 6020 Innsbruck, Austria

**Keywords:** *Neosartorya* (*Aspergillus*) *fischeri* antifungal proteins, γ-core peptide, Plant pathogenic fungus, Biofungicide, Drug combination, Synergism

## Abstract

Plant pathogenic fungi are responsible for enormous crop losses worldwide. Overcoming this problem is challenging as these fungi can be highly resistant to approved chemical fungicides. There is thus a need to develop and introduce fundamentally new plant and crop protection strategies for sustainable agricultural production. Highly stable extracellular antifungal proteins (AFPs) and their rationally designed peptide derivatives (PDs) constitute feasible options to meet this challenge. In the present study, their potential for topical application to protect plants and crops as combinatorial biofungicides is supported by the investigation of two *Neosartorya* (*Aspergillus*) *fischeri* AFPs (NFAP and NFAP2) and their γ-core PDs. Previously, the biofungicidal potential of NFAP, its rationally designed γ-core PD (γ^NFAP^-opt), and NFAP2 was reported. Susceptibility tests in the present study extended the in vitro antifungal spectrum of NFAP2 and its γ-core PD (γ^NFAP2^-opt) to *Botrytis*, *Cladosporium*, and *Fusarium* spp. Besides, in vitro additive or indifferent interactions, and synergism were observed when NFAP or NFAP2 was applied in combination with γ^NFAP^-opt. Except for γ^NFAP2^-opt, the investigated proteins and peptides did not show any toxicity to tomato plant leaves. The application of NFAP in combination with γ^NFAP^-opt effectively inhibited conidial germination, biofilm formation, and hyphal extension of the necrotrophic mold *Botrytis cinerea* on tomato plant leaves. However, the same combination only partially impeded the *B. cinerea*-mediated decay of tomato fruits, but mitigated the symptoms. Our results highlight the feasibility of using the combination of AFP and PD as biofungicide for the fungal infection control in plants and crops.

**Supplementary Information:**

The online version contains supplementary material available at 10.1007/s10526-022-10132-y.

## Introduction

Pre- and post-harvest phytopathogenic fungi cause enormous crop losses worldwide every year and threaten the increase in food supply to the human population despite the intensive agricultural application of chemical fungicides. The development of more efficient, alternative management strategies to control fungal diseases may overcome this problem (Avery et al. 2019). Alternatively, bioactive natural products for plant protection have already been used as biofungicides in sustainable agricultural production systems to reduce the impact of fungal diseases on crops. These natural compounds (e.g., phenolics, flavones, quinones, tannins, terpenes, essential oils, alkaloids, and saponins) act directly as antimicrobial agents and/or indirectly as inducers of plant defense (Gwinn 2018). Natural or rationally designed proteins and peptides from different origins with antifungal activity (van der Weerden et al. 2013) and/or with plant immunostimulatory effects (Campos et al. 2018) are also effective alternatives in an agricultural setting to fight against phytopathogenic and mycotoxigenic fungi. Primarily, they are expressed as recombinant antifungal proteins/peptides in transgenic plants to confer increased resistance to fungal pathogens (Iqbal et al. 2019). However, different international regulations for genetically modified (GM) plant breeding (Eriksson 2019) and the spread of anti-GM sentiment among the public worldwide (Tagliabue 2018) limit the application of these cultivars. The direct environmental application of antifungal proteins and peptides as topical biofungicides in plant protection has several limitations, such as a narrow antifungal spectrum, potential toxic effects on humans and animals and molecular structures that are easily degradable by extracellular proteases (Jung and Kang 2014). The rational design and the development of new formulations for the application of antimicrobial peptides might overcome these problems (Porto et al. 2012; Ajingi and Jongruja 2020). Solid-phase peptide synthesis based on 9-fluorenylmethyloxycarbonyl (Fmoc) chemistry is becoming more economic nowadays alleviating high production costs (Behrendt et al. 2016). This chemical method could provide an alternative for the industrial-scale production of antifungal proteins and peptides in the future, considering their antimicrobial activity in a low concentration range (µM) (Hegedüs and Marx 2013). Proof of concept studies previously reported on the economic production of synthetic antifungal peptides with a small chemical footprint, and their cost-effective application as microbicides in an agricultural setting (Rautenbach et al. 2016).

Further studies documented the safe applicability of recombinant small, cysteine-rich, cationic antifungal proteins of ascomycetous origins (AFPs) as biofungicides in plants and crops to fight infection with phytopathogenic filamentous fungi (Vila et al. 2001; Moreno et al. 2003, 2006; Theis et al. 2005; Barna et al. 2008; Leiter et al. 2017; Garrigues et al. 2018).

Only recently, we provided information about the biofungicidal potential and safe agricultural application of AFPs, and their rationally designed peptide derivatives (PDs). The *Penicillium chrysogenum* antifungal protein (PAF) effectively inhibited the growth of numerous pre- and post-harvest pathogenic fungi in vitro and showed no toxic effects on mammalian cells and plant seedlings (Tóth et al. 2020a). Furthermore, its topical application protected tomato plant leaves against *Botrytis cinerea* infection (Tóth et al. 2020a). Similar observations regarding the antifungal efficacy and potential agricultural applicability were reported for the *Neosartorya* (*Aspergillus*) *fischeri* antifungal protein (NFAP) and its PD (γ^NFAP^-opt), which was designed based on the evolutionary conserved antimicrobial γ-core motif of NFAP (Tóth et al. 2020b). NFAP and γ^NFAP^-opt inhibited the development of decay lesions on tomato fruits caused by *Cladosporium herbarum*, proving their feasibility as biopreservative agents in agriculture (Tóth et al. 2020b). The NFAP related *N.* (*A.*) *fischeri* antifungal protein 2 (NFAP2; Tóth et al. 2016) was detected to be moderately active in vitro against the post-harvest fungi *Penicillium digitatum* and *Penicillium italicum*, and to control *P. digitatum* infection of citrus fruit (Gandía et al. 2021).

In the present study, we extended the antifungal spectrum of NFAP2 and its rationally designed γ-core PD (γ^NFAP2^-opt) to more pre- and post-harvest plant pathogenic fungi. Additionally, we analyzed the nature of the in vitro interaction of different combinations of NFAP, NFAP2, and their respective γ-core PDs and determined their growth inhibition potential against selected plant pathogenic fungal strains. The results let hypothesize that a combination of *Neosartorya* AFP and PD, showing a synergistic interaction, can be safely administered to protect plants and crops from fungal infection, which has never been tested before. Our assumption was evidenced by the combined treatment of tomato plants infected with the necrotrophic fungal pathogen *B. cinerea* with NFAP and γ^NFAP^-opt. In this biocontrol experiment, lower effective concentrations of NFAP and γ^NFAP^-opt, when applied in combination, were sufficient to achieve the same protective effect as at higher concentrations in single use. Therefore, our study suggests that the combination of AFPs with PDs is a cost-effective biocontrol strategy and might also limit resistance development.

## Materials and methods

### Strains and media

Pre- and post-harvest plant pathogenic fungal strains used in the antifungal susceptibility tests are listed in Table 1. They were maintained on potato dextrose agar (Sigma–Aldrich, St. Louis, MO, USA) slants at 4 °C. Antifungal susceptibility tests, treatments for plant toxicity, and biocontrol assays were performed in tenfold diluted potato dextrose broth (0.1 × PDB; Sigma–Aldrich).

**Table 1:** Minimal inhibitory concentrations (µg ml^−1^) of *Neosartorya* antifungal proteins and peptide derivatives against plant pathogenic filamentous fungi (in alphabetic order)

Isolate	NFAP*	γ^NFAP^-opt*	NFAP2	γ^NFAP2^-opt	Origin of isolate
*Aspergillus flavus* SZMC 3014	100	> 200	> 200	> 200	*Triticum aestivum*/Hungary
*Aspergillus flavus* SZMC 12618	100	> 200	> 200	> 200	*Triticum aestivum*/Hungary
*Aspergillus flavus* SZMC 20745**	12.5	> 200	> 200	> 200	*Zea mays*/Hungary
*Aspergillus flavus* SZMC 20755**	25	> 200	> 200	> 200	*Zea mays*/Hungary
*Aspergillus nige*r SZMC 0145	50	> 200	> 200	> 200	Fruits/Hungary
*Aspergillus niger* SZMC 2759	50	> 200	> 200	> 200	Raisin/Hungary
*Aspergillus welwitschiae* SZMC 21821	25	> 200	> 200	> 200	*Allium cepa*/Hungary
*Aspergillus welwitschiae* SZMC 21832	12.5	> 200	> 200	> 200	*Allium cepa*/Hungary
*Botrytis cinerea* SZMC 21472**	6.25	200	50	200	*Rubus idaeus*/Hungary
*Botrytis cinerea* SZMC 21474	50	50	25	200	*Fragaria* × *ananassa*/Hungary
*Botrytis cinerea* NCAIM F.00751	50	50	12.5	12.5	Hungary
*Botrytis pseudocinerea* SZMC 21470	100	100	12.5	50	*Brassica napus*/Hungary
*Botrytis pseudocinerea* SZMC 21471	100	100	12.5	200	*Brassica napus*/Hungary
*Cladosporium herbarum* FSU 1148	100	12.5	12.5	100	n.d
*Cladosporium herbarum* FSU 969	100	12.5	12.5	100	n.d
*Fusarium boothi* CBS 110250	25	50	> 200	> 200	*Zea mays*/South Africa
*Fusarium graminearum* SZMC 6236J	25	50	> 200	> 200	Vegetables/Hungary
*Fusarium oxysporum* SZMC 6237J	25	50	50	> 200	Vegetables/Hungary
*Fusarium solani* CBS 115659	50	12.5	> 200	50	*Solanum tuberosum*/Germany
*Fusarium solani* CBS 119996	100	50	> 200	200	*Daucus carota*/The Netherlands

n.d. data not available

*According toTóth et al. (2020b)

**MIC determination for NFAP and γ^NFAP^-opt in this study

### Cultivation of tomato plants

Tomato plant seeds (*Solanum lycopersicum* L. cv. Ailsa Craig) were germinated for three days at 27 °C in the dark. Then, the seedlings were transferred to Perlite (bulk density: 90–110 kg m^−3^, particle size: 3–6 mm, moisture content: > 2%, pH = 6.0–7.5; Agrolit Kft., Olaszliszka, Hungary) for 14 days. After that, the plants were grown in a controlled environment applying 200 μmol m^−2^ s^−1^ photon flux density with a L:D 12:12 photoperiod, day/night temperatures of 23/20 °C, and RH of 55–60% for four weeks in hydroponic culture, in accordance with the work of Poór et al. (2011).

### Production of proteins and peptide derivatives

Recombinant NFAP and NFAP2 were produced in a *P. chrysogenum*-based expression system and purified by cation-exchange chromatography, as described previously (Sonderegger et al. 2016; Tóth et al. 2018). To reach maximum protein purity (100%), an additional semipreparative reverse-phase high-performance liquid chromatography step was applied after the cation-exchange chromatography, which was performed as described previously for NFAP2 (Kovács et al. 2019). The peptide γ^NFAP^-opt (Tóth et al. 2020b), the peptide spanning the NFAP2 γ-core motif (γ^NFAP2^), and its rationally designed variant (γ^NFAP2^-opt) were synthesized applying Fmoc chemistry, as described by Sonderegger et al. (2018) (Table 2).

**Table 2:** Amino acid sequences and in silico predicted physicochemical properties of the investigated *Neosartorya* antifungal proteins and peptide derivatives

Protein/peptide	Number of amino acids	Molecular weight (kDa)	Number of Cys	Number of Lys/Arg/His	Theoretical pI	Estimated charge at pH 7	GRAVY
LEYK**GECFTKDNTC**KYKIDGKTYLAKCPSAANTKCEKDGNKCTYDSYNRKVKCDFRH							
NFAP*	57	6.6	6	11/2/1	8.93	+ 5.0	− 1.214
Ac-EYKGKC(-SH)KTKKNKC(-SH)K-NH_2_							
γ^NFAP^-opt*	14	1.7	2	7/0/0	9.84	+ 5.8	− 2.264
IATSPYYACNCPNNCKHKKGSGCKYHSGPSDKSKVIS**GKCEWQGGQLNC**IAT							
NFAP2	52	5.6	6	7/0/2	9.01	+ 5.2	− 0.731
Ac-VISGKC(-SH)EWQGGQLNC(-SH)K-NH_2_							
γ^NFAP2^	16	1.8	2	2/0/0	8.02	+ 0.8	− 0.450
Ac-VISGKC(-SH)KTKKNKC(-SH)K-NH_2_							
γ^NFAP2^-opt	14	1.6	2	6/0/0	10.05	+ 5.8	− 1.079

The γ-core motif in the primary structure is indicated in bold and underlined

*GRAVY* grand average of hydropathy value, *Ac-* N-terminal acetylation, *(-SH)* free sulfhydryl group of cysteine, *-NH*_*2*_ C-terminal amidation

*According to Tóth et al. (2020b)

### In vitro antifungal susceptibility tests

In vitro antifungal susceptibility tests were performed to determine the individual minimal inhibitory concentrations (MICs) as described by Tóth et al. (2020b). The MIC was defined as the lowest AFP/PD concentration that reduces fungal growth to ≤ 5% in comparison with the untreated control which was set to be 100%. To investigate the potential synergistic interaction between the AFPs and PDs, the checkerboard microdilution method was applied (Eliopoulos and Moellering 1996). The fractional inhibitory concentration index (FICI) was calculated to reveal the nature of the interaction, as described by Pillai et al. (2005). The interaction between the two compounds was considered as synergistic (FICI ≤ 0.5), indifferent or additive (0.5 < FICI ≤ 4.0), or antagonistic (FICI ≥ 4.0) (Pillai et al. 2005). Susceptibility tests were prepared in three technical replicates and repeated two times.

### Plant toxicity assay

AFPs and PDs were dissolved in sterile distilled water and dropped in 10 µl aliquots at five points between the left lateral veins of the abaxial leaf epidermis from fully expanded leaves of the second leaf level of tomato plants. The following concentrations were applied: 12.5 µg ml^−1^ NFAP, 400 µg ml^−1^ γ^NFAP^-opt, 100 µg ml^−1^ NFAP2, and 400 µg ml^−1^ γ^NFAP2^-opt. The plants were then kept in a humid (60%) plant growth chamber for three weeks at 23 °C under photoperiodic day–night simulation (12/12 h with or without illumination at 1200 lx). After this treatment, the leaves were detached and Evan’s blue staining was applied to visualize the necrotic zone around the treatment points, as reported by Tóth et al. (2020b). The stained leaves were photographed with a Canon EOS 700D camera (Tokyo, Japan). Two branches each with five leaves of two plants for each treatment were used in one experiment. Plant toxicity assays were repeated two times.

### Scanning electron microscopy (SEM)

SEM experiments were performed on tomato plant leaves. In this set-up, *B. cinerea* SZMC 21472 conidia (10^7^ conidia ml^−1^) were mixed with NFAP (6.25 µg ml^−1^ and 1.56 µg ml^−1^) or γ^NFAP^-opt (200 µg ml^−1^ and 6.25 µg ml^−1^). In combinatorial assays, NFAP and γ^NFAP^-opt were applied together at the concentration of 1.56 µg ml^−1^ and 6.25 µg ml^−1^, respectively. Ten microliters of this suspension were spotted on three points between the lateral veins of the abaxial leaf epidermis. Leaves treated with conidial suspension without protein/peptide were used as infection controls. Untreated leaves served as uninfected controls. The leaves were placed in Petri dishes containing three sterilized filter papers (0113A00009 qualitative filter paper; Filters Fioroni, Ingré, France) wetted with sterile ddH_2_O. These Petri dishes were kept in a humid (60%) plant growth chamber at 23 °C under photoperiodic day-night simulation (12/12 h with or without illumination at 1200 lx). After incubation for four days, the infected leaf zones around the conidial spots were clipped out, and fixed with 2.5% (v/v) glutaraldehyde and 0.05 M cacodylate buffer (pH = 7.2) in phosphate buffered saline (PBS; pH = 7.4) overnight at 4 °C. Then, they were washed twice with PBS and dehydrated with a graded ethanol series [30%, 50%, 70%, 80% (v/v)] for 2 h each at room temperature, and stored in 100% (v/v) ethanol overnight at 4 °C. The discs were dried with a Quorum K850 critical-point dryer (Quorum Technologies, Laughton, UK), followed by 12 nm gold coating, and observed under a JEOL JSM-7100F/LV field emission scanning electron microscope (JEOL Ltd., Tokyo, Japan). Three leaves for each treatment were analyzed and the experiment was repeated two times.

### Plant and crop protection assays

Plant and crop protection assays were performed on detached *B. cinerea* SZMC 21472-infected tomato plant leaves and on tomato fruits, respectively, in accordance with the procedures reported by Tóth et al. (2020a, b). The protective effect of NFAP (6.25 µg ml^−1^ and 1.56 µg ml^−1^) and γ^NFAP^-opt (200 µg ml^−1^ and 6.25 µg ml^−1^) was tested. In combinatorial assays, NFAP and γ^NFAP^-opt were applied together at the concentration of 1.56 µg ml^−1^ and 6.25 µg ml^−1^, respectively. The incidence of the infection was calculated in percentage. Statistical analyses were performed using the Statistics Kingdom online platform to calculate Levene’s, one-way ANOVA, and Tukey’s HSD post-hoc tests (Statistics Kingdom 2021). Plant protection assays were repeated three times involving three technical replicates, while the crop protection assays were repeated two times involving three technical replicates.

## Results

### In vitro antifungal potential of *N. fischeri* AFPs and their PDs against pre- and post-harvest plant pathogenic fungi

In the present study, we investigated the antifungal activity of NFAP2 and its rationally designed PD γ^NFAP2^-opt. For comparative purposes, the data of this study are summarized with the data reported by Tóth et al. (2020b) in Table 1. NFAP2 showed high growth inhibitory efficacy against *Botrytis* (MIC = 12.5–50 µg ml^−1^) and *Cladosporium* isolates (MIC = 12.5 µg ml^−1^). However, none of the tested aspergilli and fusaria were susceptible in the investigated protein concentration range. The PD spanning the C-terminal γ-core motif of NFAP2 (γ^NFAP2^ in Table 2), had no antifungal activity (data not shown). However, the rationally designed PD γ^NFAP2^-opt (Table 2) was more positively charged and hydrophilic and inhibited the growth of *Botrytis*, *Cladosporium*, and *Fusarium* isolates at MICs ranging between 12.5 and 200 µg ml^−1^.

### Toxicity of *N. fischeri* AFPs and their PDs on plant leaves

**Fig. 1:**
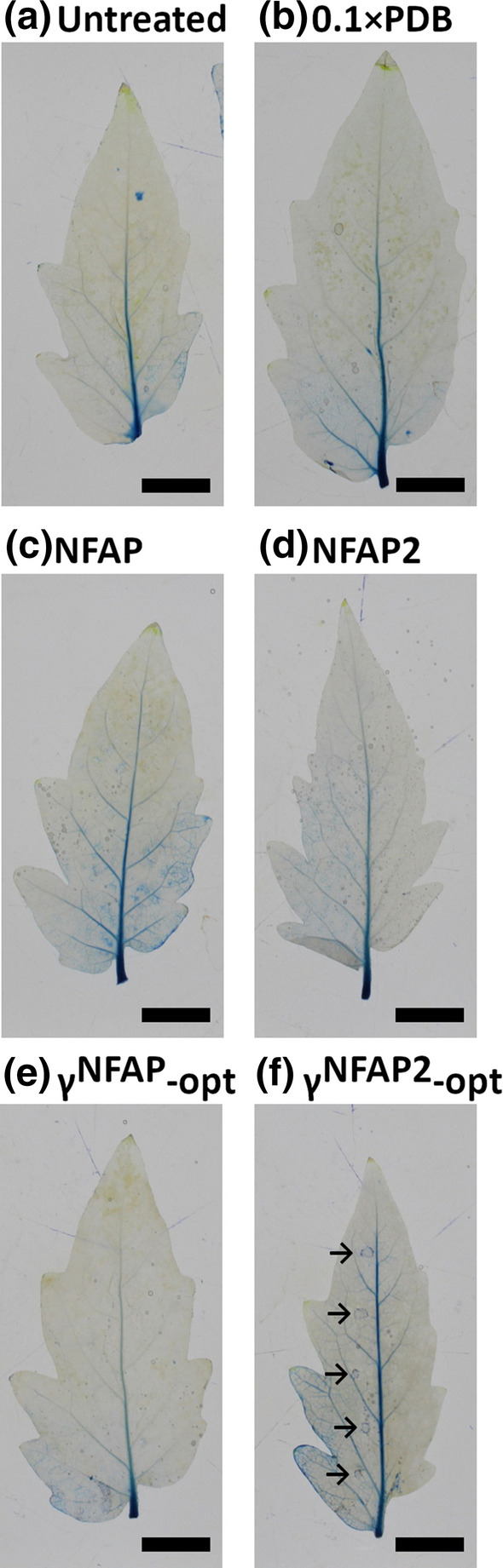
Evan’s blue staining of tomato plant leaves to monitor cytotoxic effects of *Neosartorya fischeri* NRRL 181 antifungal proteins and their peptide derivatives. The leaves were treated with 10 µl aliquots of NFAP (12.5 µg ml^−1^) (**c**), NFAP2 (100 µg ml^−1^) (**d**), γ^NFAP^-opt (400 µg ml^−1^) (**e**), and γ^NFAP2^-opt (400 µg ml^−1^) (**f**) and the appearance of necrotic areas was compared with that of control leaves left untreated (**a**) or treated with 10 µl of 0.1 × PDB (**b**). Blue-colored zones (marked by black arrows) indicate cell death at the treatment points. Scale bars represent 1 cm. (Color figure online)

The potential toxic effects of antifungal active *N. fischeri* AFPs and PDs were investigated on intact tomato plant leaves at concentrations twofold higher than the respective MIC detected in vitro against the necrotrophic fungal pathogen *B. cinerea* SZMC 21472. The applied Evan’s blue staining (Vijayaraghavareddy et al. 2017), which is an appropriate method to monitor necrotic areas at the treatment points, did not indicate any plant cell killing ability of NFAP, NFAP2, and γ^NFAP^-opt (Fig. 1). However, the area where γ^NFAP2^-opt was applied stained blue was indicative for plant cell cytotoxicity (Fig. 1). Therefore, this PD was excluded from further experiments.

### Antifungal activity of *N. fischeri* AFPs and their PDs in combination

The checkerboard titration method was applied to reveal the nature of the interaction when *Neosartorya* AFPs (NFAP and NFAP2) were combined with each other or with γ^NFAP^-opt, against *Aspergillus flavus* SZMC 20745, *B. cinerea* SZMC 21472, *Cladosporium herbarum* FSU 1148, and *Fusarium oxysporum* SZMC 6237J (Table 3). Based on the FICI values, the NFAP + γ^NFAP^-opt combination showed a synergistic interaction against *B. cinerea* SZMC 21472 (FICI = 0.28) and *C. herbarum* FSU 1148 (FICI = 0.31), respectively. Other combinations of AFPs and γ^NFAP^-opt resulted in additive or indifferent interactions on these two plant pathogens (FICI = 1.25–1.50). The same additive or indifferent interaction was found with NFAP2 + γ^NFAP^-opt on *F. oxysporum* SZMC 6237J. Notably, no MICs could be determined with *A. flavus* SZMC 20745 upon exposure to the combinations NFAP + NFAP2, NFAP + γ^NFAP^-opt, and NFAP2 + γ^NFAP^-opt, and with *F. oxysporum* SZMC 6237J treated with NFAP + NFAP2 and NFAP + γ^NFAP^-opt. The NFAP + NFAP2 and NFAP + γ^NFAP^-opt combinations resulted in a so-called paradoxical growth effect at these two fungi, namely, they resumed growth when treated with AFP/PD concentrations above the individual MICs. No antagonistic interaction was observed for any of the tested combinations. These data are summarized in Table 3.

**Table 3:** Checkerboard titration results of *Neosartorya* antifungal proteins and γNFAP-opt peptide against pre- and post-harvest pathogenic fungi based on the fractional inhibitory concentration index (FICI) values

	*Aspergillus flavus* SZMC 20745	*Botrytis cinerea* SZMC 21472	*Cladosporium herbarum* FSU 1148	*Fusarium oxysporum* SZMC 6237J	
NFAP + NFAP2					
NFAP (MIC)	12.5	6.25	100	25	MIC (µg ml^−1^)
NFAP2 (MIC)	> 200	50	12.5	50	
NFAP (MIC_comb_)	> 25	6.25	100	> 50	
NFAP2 (MIC_comb_)	> 200	12.5	3.125	> 50	
FICI	–	1.25	1.25	–	
Type of the interaction	–*	Additive or indifference	Additive or indifference	–*	
NFAP + γ^NFAP^-opt					
NFAP (MIC)	12.5	6.25	100	25	MIC (µg ml^−1^)
γ^NFAP^-opt (MIC)	> 200	200	12.5	50	
NFAP (MIC_comb_)	> 25	1.56	100	> 25	
γ^NFAP^-opt (MIC_comb_)	> 200	6.25	6.25	> 50	
FICI	–	0.28	1.50	–	
Type of the interaction	–*	Synergy	Additive or indifference	–*	
NFAP2 + γ^NFAP^-opt					
NFAP2 (MIC)	> 200	50	12.5	50	MIC (µg ml^−1^)
γ^NFAP^-opt (MIC)	> 200	200	12.5	50	
NFAP2 (MIC_comb_)	> 200	12.5	0.78	50	
γ^NFAP^-opt (MIC_comb_)	> 200	200	3.125	12.5	
FICI	–	1.25	0.31	1.25	
Type of the interaction	–	Additive or indifference	Synergy	Additive or indifference	

MIC and MIC_comb_ MIC of antifungal protein/peptide when applied alone and in combination, respectively. Type of the interaction: 0.5 ≤ FICI ≤ 4.0: additive or indifference, FICI < 0.5: synergy, FICI > 4.0: antagonism (Pillai et al. 2005)

*paradoxical effect

### Biocontrol efficacy of NFAP, γ^NFAP^-opt and their synergistic combination

Based on the synergistic activity of NFAP and γ^NFAP^-opt on *B. cinerea* and of NFAP2 and γ^NFAP^-opt on *C. herbarum *in vitro (Table 3), we hypothesized that a combination of specific AFPs and PDs should have a remarkable biocontrol activity and allow the reduction of the antifungal effective dosage of both compounds to reach the same protection against fungal infection as the single application at their individual MICs. To provide a proof of principle, we tested this assumption in biocontrol assays and characterized the antifungal efficacy of the combination of NFAP and γ^NFAP^-opt on tomato plants against the infection with *B. cinerea,* the most common necrotrophic pathogen of above-ground parts of this plant (Nambeesan et al. 2012).

**Fig. 2:**
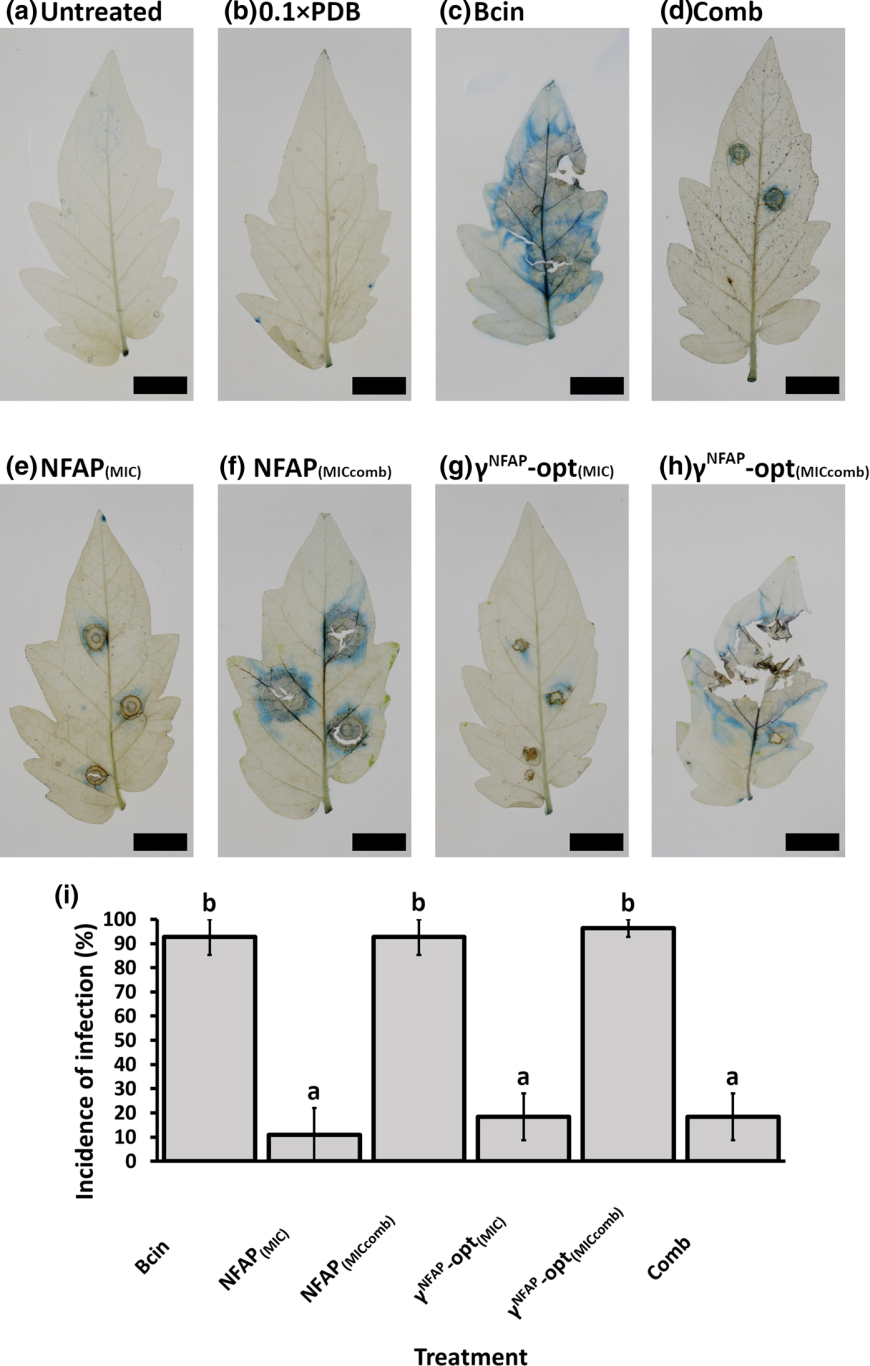
Evan’s blue staining of necrotic plant tissue on tomato plant leaves after *Botrytis cinerea* SZMC 21472 infection in comparison with the uninfected control (**a**). Leaves were treated with 0.1 × PDB (**b**), *B. cinerea* (Bcin) (**c**), *B. cinerea* + synergistic combination of NFAP and γ^NFAP^-opt (Comb: 1.56 and 6.25 µg ml^−1^, respectively) (**d**), *B. cinerea* + MIC of NFAP (NFAP_(MIC)_: 6.25 µg ml^−1^) (**e**), *B. cinerea* + 1.56 µg ml^−1^ NFAP (NFAP_(MICcomb)_) (**f**), *B. cinerea* + MIC of γ^NFAP^-opt (γ^NFAP^-opt_(MIC)_: 200 µg ml^−1^) (**g**), *B. cinerea* + 6.25 µg ml^−1^ γ^NFAP^-opt (γ^NFAP^-opt_(MICcomb)_) (**h**). Blue-colored zones indicate cell death at the treatment points. Scale bars represent 1 cm. (**i**) Incidence of *B. cinerea* SZMC 21472 infection on the treated leaves in comparison with the untreated controls (Bcin). Bars represent the mean ± SE of developed infection at treatment points (n = 3). a: significant difference (p < 0.001) in comparison with infected, untreated leaves (Bcin). b: significant difference (p < 0.001) in comparison with leaves that were infected and treated with synergistic NFAP and γ^NFAP^-opt combination (Comb). (Color figure online)

Detached tomato plant leaves were infected with *B. cinerea* SZMC 21472 conidia and treated with NFAP or γ^NFAP^-opt at their MIC (6.25 µg ml^−1^ and 200 µg ml^−1^, respectively), NFAP or γ^NFAP^-opt at concentrations below the MIC (1.56 µg ml^−1^ and 6.25 µg ml^−1^, respectively), the synergistic combination of NFAP (1.56 µg ml^−1^) and γ^NFAP^-opt (6.25 µg ml^−1^), and the incidence of infection was compared to the infected, but untreated control. ANOVA showed that there was a significant difference between the incidences of infections at the six different treatments (F_5, 12_ = 25.58, p < 0.001). In leaves that were infected but left untreated, the appearance of necrotic lesions and intensive Evan’s blue staining around the infection areas indicated the severe destruction of tomato plant leaf tissues by *B. cinerea* (Bcin in Fig. 2c). The application of NFAP or γ^NFAP^-opt at their MIC (6.25 µg ml^−1^ and 200 µg ml^−1^, respectively) protected the leaves against this fungal pathogen: necrotic lesions and intensive blue staining were not observed under these experimental conditions (NFAP_(MIC)_ and γ^NFAP^-opt_(MIC)_ in Fig. 2e and g). The incidence of infection was significantly lower (p < 0.001 according to the Tukey’s HSD post-hoc test) compared to the infected leaves that were left untreated (Fig. 2i). The application of NFAP or γ^NFAP^-opt at concentrations below the MIC was not effective (NFAP_(MICcomb)_ and γ^NFAP^-opt_(MICcomb)_ in Fig. 2i). NFAP mitigated the symptoms of *B. cinerea* infection at a concentration of 1.56 µg ml^−1^ by reducing the area of tissue destruction, but could not fully protect the leaves from infection (NFAP_(MICcomb)_ in Fig. 2f). In contrast, 6.25 µg ml^−1^ γ^NFAP^-opt did not prevent the development of infection: extensive necrotic lesions and blue-colored zones were visible at the points of *B. cinerea* inoculation (γ^NFAP^-opt_(MICcomb)_ in Fig. 2h). Notably, the synergistic combination of NFAP (1.56 µg ml^−1^) and γ^NFAP^-opt (6.25 µg ml^−1^) significantly (p < 0.001 according to the Tukey’s HSD post-hoc test) reduced the invasion of the fungus into the leaf tissue and protected tomato plant leaves against *B. cinerea* infection (Comb in Fig. 2d and i). According to Tukey’s HSD post-hoc test, there was no significant difference in the efficacy between the protective effects of Comb and NFAP_(MIC)_ (p = 0.988), Comb and γ^NFAP^-opt_(MIC)_ (p = 1.000), NFAP_(MIC)_ and γ^NFAP^-opt_(MIC)_ (p = 0.988). The above results indicated that combined application of NFAP and γ^NFAP^-opt allowed the reduction of their effective dosage to achieve the same protective effect against *B. cinerea* infection as high concentrations of these compounds in single use.

**Fig. 3:**
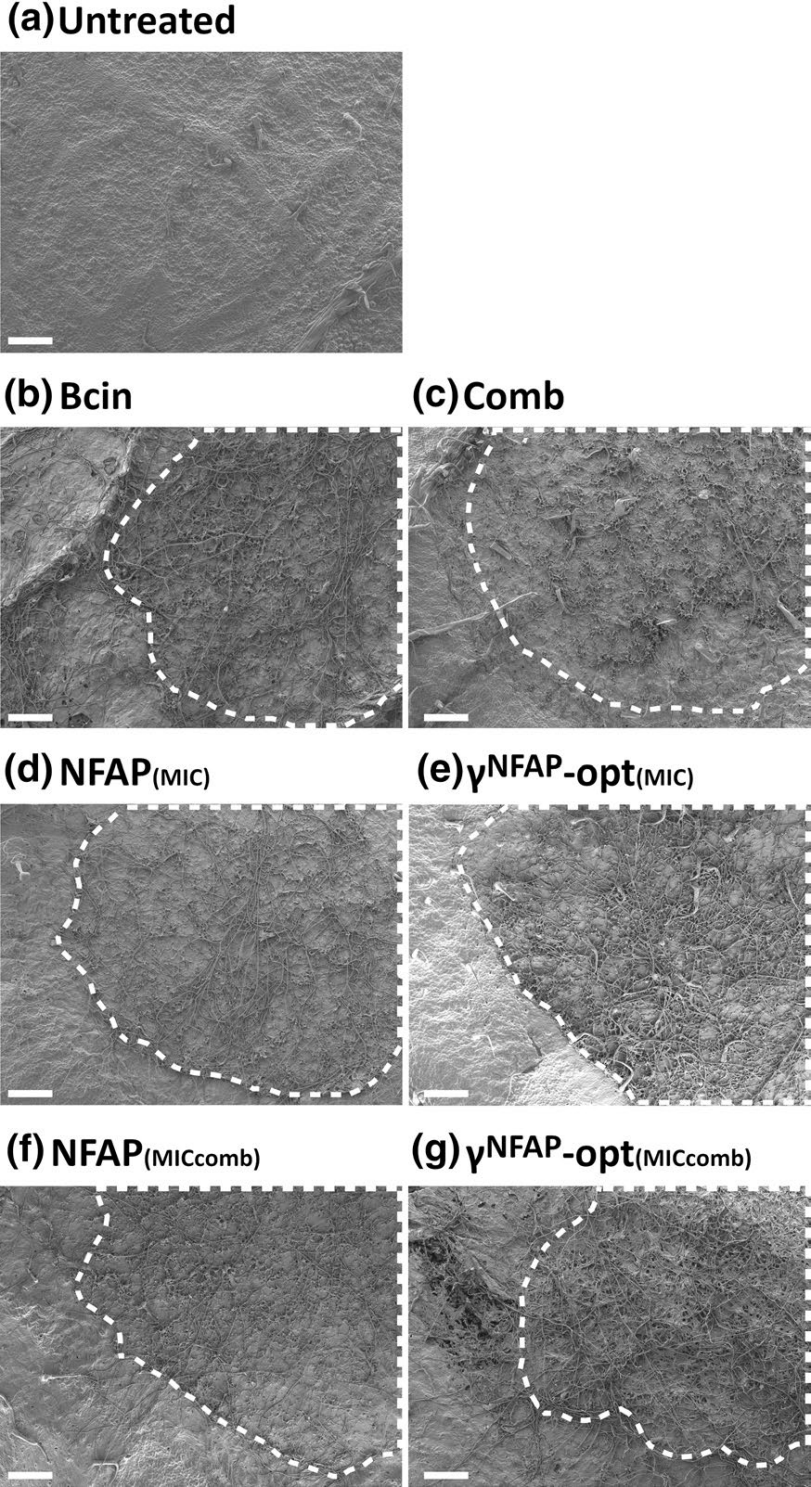
Scanning electron microscopy of *Botrytis cinerea* SZMC 21472 infection on tomato plant leaves after treatment with the combination of NFAP and γ^NFAP^-opt (Comb: 1.56 and 6.25 µg ml^−1^, respectively) (**c**), at their MIC (NFAP_(MIC)_: 6.25 µg ml^−1^ (**d**); γ^NFAP^-opt_(MIC)_: 200 µg ml^−1^ (**e**), with 1.56 µg ml^−1^ NFAP (NFAP_(MICcomb)_) (**f**), and with 6.25 µg ml^−1^ γ^NFAP^-opt (γ^NFAP^-opt_(MICcomb)_) (**g**) in comparison with the uninfected/untreated and infected/untreated controls (untreated (**a**) and Bcin (**b**), respectively). The infection areas and treatment areas are framed with a white dashed line. Scale bars represent 200 µm. (Color figure online)

**Fig. 4:**
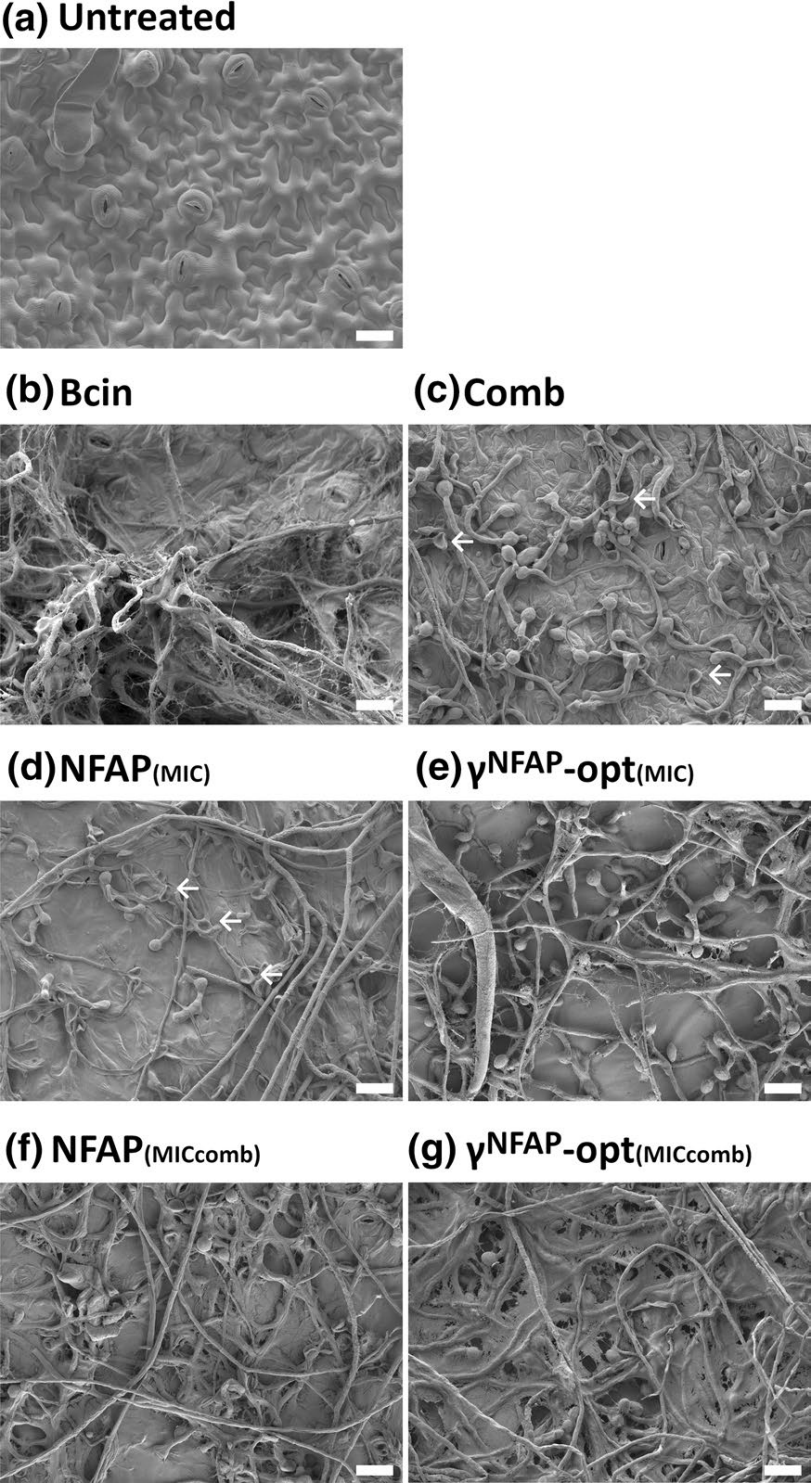
Scanning electron microscopy of *Botrytis cinerea* SZMC 21472 infection on tomato plant leaves after treatment with the combination of NFAP and γ^NFAP^-opt (Comb: 1.56 and 6.25 µg ml^−1^, respectively) (**c**), at their MIC (NFAP_(MIC)_: 6.25 µg ml^−1^ (**d**); γ^NFAP^-opt_(MIC)_: 200 µg ml^−1^ (**e**), with 1.56 µg ml^−1^ NFAP (NFAP_(MICcomb)_) (**f**), and with 6.25 µg ml^−1^ γ^NFAP^-opt (γ^NFAP^-opt_(MICcomb)_) (**g**) in comparison with the uninfected/untreated and infected/untreated controls (untreated (**a**) and Bcin (**b**), respectively). White arrows indicate examples for destroyed germlings. Scale bars represent 20 µm. (Color figure online)

Next, we visualized the infection of leaves with *B. cinerea* and the protective effect of NFAP and γ^NFAP^-opt by SEM. The results indicated that without any treatment, the hyphae colonized the leaf surface forming a dense mycelium, which spread out beyond the inoculation area (Bcin in Fig. 3b). The application of NFAP at its MIC (6.25 µg ml^−1^) and at the lower concentration of 1.56 µg ml^−1^ reduced the dispersion of the fungal infection from the treatment areas (NFAP_(MIC)_ and NFAP_(MICcomb)_ in Fig. 3d and f, respectively). The same was true for γ^NFAP^-opt when applied at its MIC (200 µg ml^−1^) (γ^NFAP^-opt_(MIC)_ in Fig. 3e), while at lower concentration (6.25 µg ml^−1^) this peptide was not effective enough to inhibit the colonization of the leaf surface beyond the treatment area (γ^NFAP^-opt_(MICcomb)_ in Fig. 3g). The synergistic combination of NFAP and γ^NFAP^-opt (1.56 µg ml^−1^ and 6.25 µg ml^−1^, respectively) significantly reduced the colonization and hyphal extension of *B. cinerea* SZMC 21472 on the leaf (Comb in Fig. 3c). The SEM analysis further revealed that *B. cinerea* SZMC 21472 established a biofilm on the leaves which consisted of several layers of well-developed hyphae (Bcin in Fig. 4b). When applied at their MIC, NFAP (6.25 µg ml^−1^) and γ^NFAP^-opt (200 µg ml^−1^) destroyed most of the conidia and germlings, and reduced biofilm formation (NFAP_(MIC)_ and γ^NFAP^-opt_(MIC)_ in Fig. 4d and e, respectively). At concentrations lower than the respective MIC, NFAP (1.56 µg ml^−1^) and γ^NFAP^-opt (6.25 µg ml^−1^) did not prevent the germination of conidia and the initiation of biofilm formation (NFAP_(MICcomb)_ and γ^NFAP^-opt_(MICcomb)_ in Fig. 4f and g, respectively). The synergistic combination of NFAP (1.56 µg ml^−1^) and γ^NFAP^-opt (6.25 µg ml^−1^), however, remarkably reduced the germination ability of *B. cinerea* SZMC 21472 conidia, and hampered the formation of a biofilm (Comb in Fig. 4c).

Finally, we tested the biocontrol efficacy of NFAP, γ^NFAP^-opt and their combination in the protection of tomato fruits from fungal infection. None of the treatments applied fully protected tomato fruits from *B. cinerea*-caused decay*.* However, the treatment with the MIC of NFAP or γ^NFAP^-opt and their synergistic combination decreased the fungal spread on the fruit surface (data not shown).

## Discussion

There is an urgent need to develop new antifungal treatment strategies in order to counteract the enormous crop losses due to fungal infection and contamination, and to support the increase in global calorie consumption in the coming decades. In the present study, we further evidenced the potential applicability of AFPs of ascomycetous origin and their rationally designed PDs for protecting plants and crops from infection with phytopathogenic fungi.

In our previous studies, we reported that PAF from *P. chrysogenum* and NFAP from *N. fischeri* inhibit the growth of several pre- and post-harvest plant pathogenic fungi in vitro, and they differed in their antifungal spectrum and efficacy (Tóth et al. 2020a, b). We observed that these features are highly dependent on the amino acid composition of the evolutionary conserved γ-core motif (consensus sequence GXC-X_[3–9]_-C) of the protein, in which X can be any amino acid (Sonderegger et al. 2018). Increasing the positive charge and hydrophilicity of this motif by amino acid substitutions elevated the antifungal efficacy of PAF against yeasts (Sonderegger et al. 2018), and changed its antifungal spectrum on phytopathogenic molds (Tóth et al. 2020a). These results led us to assume that the antifungal activity of AFPs is at least in part regulated by the γ-core region. This assumption was further supported by the observation that short synthetic peptides spanning the γ-core region of PAF and NFAP are antifungal active and that their efficacy is increased by elevating the positive net charge (Sonderegger et al. 2018; Tóth et al. 2020a, b). NFAP2 is primarily known as an anti-yeast AFP (Tóth et al. 2016, 2018). However, our recent study provided information about its growth inhibitory activity on post-harvest pathogenic *Penicillium* spp. (Gandía et al. 2021). Here we extended the antifungal spectrum of NFAP2 to other plant pathogenic fungi, such as *B. cinerea*, *B. pseudocinerea*, *C. herbarum*, and *F. oxysporum* (Table 1). Our results clearly indicate that NFAP2 is not solely a yeast-specific AFP, as we previously supposed. Unsurprisingly, the NFAP2-derived PD, spanning the native γ-core motif (γ^NFAP2^ in Table 2), did not inhibit fungal growth, but its rationally designed variant with elevated positive charge and increased hydrophilicity (γ^NFAP2^-opt in Table 2) showed remarkable antifungal activity (Table 1). These results strengthen our previous observations regarding the features of γ-core PDs of PAF and NFAP, namely, that a high positive net charge improves the antifungal efficacy (Sonderegger et al. 2018; Tóth et al. 2020a, b).

The agricultural application of AFPs and PDs requires their good tolerance in plants and mammals. We already proved that NFAP (Tóth et al. 2020b) and NFAP2 (Kovács et al. 2019) are non-toxic to mammalian cell lines. The rationally designed γ-core PDs with high positive net charge and low hydrophilicity may have adverse effects on the viability of mammalian cells (Tóth et al. 2020b). Evan’s blue staining indicated that NFAP and NFAP2 are non-toxic to plants (Fig. 1), similar to the results obtained for PAF and its γ-core optimized protein variant (Tóth et al. 2020a). This was also true for the highly hydrophilic PD γ^NFAP^-opt. Although the net charge of γ^NFAP2^-opt is similar to that of γ^NFAP^-opt (Table 2), it is less hydrophilic and negatively affects plant cells (Fig. 1). One might speculate that the potential toxicity of short γ-core PDs on plant cells depends on their overall hydrophobicity.

The combinatorial application of antifungal compounds with different modes of action is considered when the infective fungus shows low susceptibility or resistance to one of these molecules, and/or prolonged administration of a single drug at a high dosage is toxic to the host or promotes the development of resistance (Hill and Cowen 2015). In case that there is a synergistic or additive interaction of two antifungal compounds, their co-administration allows a reduction in the effective dosage for successful therapy. It may also shorten the treatment period, decrease the risk of toxic effects in the host, and minimize the potential of the fungus to develop resistance (Belanger et al. 2015). Therefore, we investigated in the present study the in vitro interaction between *Neosartorya* AFPs and PDs and the efficacy of their combined application for protecting plants and crops against *B. cinerea* infection. The successful combination of *Aspergillus giganteus* AFP and the insect-derived antifungal peptide cecropin A against *B. cinerea* was reported by Moreno et al. (2003), who observed an additive effect of these two compounds in vitro in combinatorial titration assays. In agreement with this finding, an additive effect was detected with the two *Neosartorya* AFPs (NFAP and NFAP2) (Table 3). However, we proved that NFAP and NFAP2 synergistically interact with the rationally designed PD γ^NFAP^-opt in vitro (Table 3). Similarly to these results, in vitro synergistic interactions between PAF and PDs derived from the *P. digitatum* antifungal protein B (AfpB) against the post-harvest mold *P. digitatum*, and between PAF and a rationally designed antifungal hexapeptide (PAF 26) against *P. digitatum* and *Aspergillus niger* were documented by Garrigues et al. (2017).

The observed synergistic interaction between NFAP and γ^NFAP^-opt against *B. cinerea* could result from their different modes of action or cellular targets. NFAP induces apoptosis in *Aspergillus fumigatus* via a heterotrimeric G-protein signaling pathway (Virágh et al. 2015), or by binding to an intracellular target in *Neurospora crassa* after its internalization by an energy-dependent uptake mechanism (Hajdu et al. 2019). Annexin V-FITC/propidium iodide staining revealed that NFAP triggers apoptosis that results in necrosis in *B. cinerea* conidia after a 16 h incubation, whereas γ^NFAP^-opt is a membrane-acting peptide that does not induce apoptosis, but readily (4 h incubation) disrupts the outer layers of *B. cinerea* conidia (Supplementary Fig. S1). The observed synergism between NFAP and γ^NFAP^-opt suggests that killing of fungal pathogens by their combination results from different antifungal mechanisms.

The synergistic activity of NFAP and γ^NFAP^-opt administered in combination in vitro and in the biocontrol experiments was clearly detectable. The bioassays evidenced that the combination of reduced dosages of NFAP and γ^NFAP^-opt protected tomato plant leaves against *B. cinerea* infection as effectively as their application alone at their MICs (Figs. 2 and 3). More importantly, this synergistic activity inhibited the ability of *B. cinerea* to form a biofilm on detached tomato plants leaves (Comb in Fig. 4), which was unambiguously documented by SEM analysis (Bcin in Fig. 4). This parallels previous descriptions of *B. cinerea* growing in heavily layered extensive hyphal networks embedded in an extracellular polymeric substance matrix on tomato stems (Harding et al. 2010). Biofilm formation of plant pathogenic fungi plays a critical role in the pathogenesis of plant diseases, and underlines the need for developing novel plant disease management strategies (Villa et al. 2017).

Recently, we demonstrated for the first time in fruit protection experiments that combinations of AFPs of different fungal origin (such as *P. chrysogenum* antifungal protein B from *P. chrysogenum*, *Penicillium expansum* antifungal protein A from *P. expansum*, and NFAP2 from *N. fischeri*) did not improve the efficacy to protect orange and apple fruits from infection with the postharvest molds *P. digitatum* and *P. expansum* compared to single treatments (Gandía et al. 2021). We observed also in the present study that the application of a synergistic combination of NFAP and γ^NFAP^-opt did not fully impede the tomato fruit decay. However, it remarkably inhibited the extension of *B. cinerea* infection on the fruit surface (data not shown).

Taken together, our findings demonstrated that NFAP and γ^NFAP^-opt reduced biofilm formation on plant surfaces and crop decay by the phytopathogenic mold *B. cinerea* when topically applied in combination. The synergistic interaction of this AFP and PD allowed their administration at lower concentrations than their MICs in single dosage. In this study we provided new insights into the biocontrol potential of AFPs and PDs, which promise the development of new protection strategies against phytopathogenic fungi.

## Supplementary Information

Below is the link to the electronic supplementary material.

Supplementary file1 (DOCX 81 KB)

## Data Availability

Data and materials are available upon request.
