# Supplementary material for: The combination of Neosartorya (Aspergillus) fischeri antifungal proteins with rationally designed γ-core peptide derivatives is effective for plant and crop protection
Source: Biocontrol (Dordr). 2022 Feb 4;67(2):249–62. doi: 10.1007/s10526-022-10132-y (PMC8993730; doi:10.1007/s10526-022-10132-y)
Supplement: Supplementary file 1 — Supplementary file1 (DOCX 81 KB) [file 10526_2022_10132_MOESM1_ESM.docx]

Supplementary Information 1


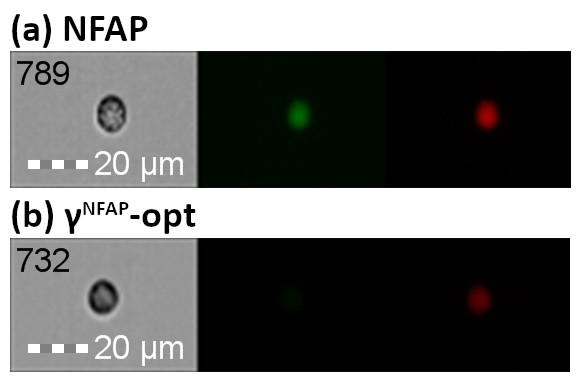


**Fig. S1** Staining of *Botrytis cinerea* SZMC 21472 conidia with Annexin V-FITC Apoptosis Detection kit (Sigma–Aldrich) after treatment with NFAP (MIC: 6.25 µg ml^-1^) (**a**), and γ^NFAP^-opt (MIC: 200 µg ml^-1^) (**b**) for 16 and 4 h, respectively, at 25°C under shaking at 160 rpm. Green fluorescence (V-FITC-positive) indicates apoptotic, while red (propidium iodide-positive) fluorescence shows necrotic conidia. Healthy conidia do not show fluorescent signal. Annexin V-FITC and PI-positive conidia were detected and pictures were taken by a FlowSight imaging flow cytometer (Amins; Merck Millipore, Billerica, MA, USA). Propidium iodide is a membrane-impermeable, red-fluorescent nuclear, and chromosome stain. It stains the conidia red if the cell membrane is disrupted. Annexin V-FITC binds phosphatidylserine on the membrane surface, when this is translocated from the inner leaflet to the external leaflet during apoptosis. Scale bars represent 20 µm.
